# Interleukin-15 Modulates Adipose Tissue by Altering Mitochondrial Mass and Activity

**DOI:** 10.1371/journal.pone.0114799

**Published:** 2014-12-17

**Authors:** Nicole G. Barra, Rengasamy Palanivel, Emmanuel Denou, Marianne V. Chew, Amy Gillgrass, Tina D. Walker, Josh Kong, Carl D. Richards, Manel Jordana, Stephen M. Collins, Bernardo L. Trigatti, Alison C. Holloway, Sandeep Raha, Gregory R. Steinberg, Ali A. Ashkar

**Affiliations:** 1 Department of Pathology and Molecular Medicine and McMaster Immunology Research Centre, McMaster University, Hamilton, Ontario, Canada; 2 Institute for Infectious Disease Research, McMaster University, Hamilton, Ontario, Canada; 3 Department of Medicine and Division of Endocrinology and Metabolism, McMaster University, Hamilton, Ontario, Canada; 4 Department of Medicine and Farncombe Family Digestive Health Research Institute, McMaster University, Hamilton, Ontario, Canada; 5 Department of Biochemistry and Biomedical Sciences and Atherothrombosis Research Group, McMaster University, Hamilton, Ontario, Canada; 6 Department of Obstetrics and Gynecology, McMaster University, Hamilton, Ontario, Canada; 7 Department of Pediatrics, McMaster University, Hamilton, Ontario, Canada; Dasman Diabetes Institute, Kuwait

## Abstract

Interleukin-15 (IL-15) is an immunomodulatory cytokine that affects body mass regulation independent of lymphocytes; however, the underlying mechanism(s) involved remains unknown. In an effort to investigate these mechanisms, we performed metabolic cage studies, assessed intestinal bacterial diversity and macronutrient absorption, and examined adipose mitochondrial activity in cultured adipocytes and in lean IL-15 transgenic (IL-15tg), overweight IL-15 deficient (IL-15−/−), and control C57Bl/6 (B6) mice. Here we show that differences in body weight are not the result of differential activity level, food intake, or respiratory exchange ratio. Although intestinal microbiota differences between obese and lean individuals are known to impact macronutrient absorption, differing gut bacteria profiles in these murine strains does not translate to differences in body weight in colonized germ free animals and macronutrient absorption. Due to its contribution to body weight variation, we examined mitochondrial factors and found that IL-15 treatment in cultured adipocytes resulted in increased mitochondrial membrane potential and decreased lipid deposition. Lastly, IL-15tg mice have significantly elevated mitochondrial activity and mass in adipose tissue compared to B6 and IL-15−/− mice. Altogether, these results suggest that IL-15 is involved in adipose tissue regulation and linked to altered mitochondrial function.

## Introduction

Current global obesity statistics are staggering - age-standardized prevalence has nearly doubled from 6.4% in 1980 to 12.0% in 2008, amounting to over 600 million individuals classified as obese [1]. This international epidemic is especially alarming not only due to the unprecedented rate by which incidence of obesity is increasing, but because obesity leads to the development of numerous chronic conditions such as diabetes, heart disease, osteoarthritis, mental illness, sleep apnea, and some forms of cancer [1]–[3]. With rising rates of childhood obesity and its associated co-morbidities, obesity represents a tremendous burden on the health of succeeding generations [3]–[5]. Therefore, understanding underlying causes and mechanisms of this disease is critical for preventing obesity from continuing to overwhelm international health care systems.

Simply monitoring an individual's caloric intake and/or activity level is not an effective treatment strategy for obesity since a variety of factors may contribute to the manifestation of this disease [6]. Intriguing research has recently emerged examining the role of intestinal microorganisms in energy homeostasis and obesity. Variations in gut bacterial diversity exist between obese and lean individuals, suggesting differences in digestion and absorption could be important factors in the pathogenesis of obesity [7]–[9]. Reports have shown that obese humans, diet induced obese and genetically obese mice have significantly fewer Bacteriodetes and an increase in Firmicutes phyla compared to lean controls [7]–[9]. Research has shown that early differences in gut microbiota in children followed from the first year to 7 years of life correlates with an overweight or obese status [10]. Lastly, dietary weight loss in humans correlates with altered gut flora, characterized by an increase in Bacteriodetes and decrease in Firmicutes phyla [9]. These reports suggest strategies targeting the modulation of the gut microbiota may provide unique treatments for obesity. These findings challenge the classical understanding of the causes of obesity and make obvious the need to better understand the relationship between the gut microbiota and obesity.

Another recently described observation that may contribute to the obesogenic state is mitochondrial dysfunction in host metabolic tissues. Mitochondria are organelles within nucleated cells involved in producing energy in the form of adenosine triphosphate (ATP) molecules through the oxidation of macronutrients including fatty acids. It is believed that the majority of the body's daily ATP needs are produced by beta oxidation of fatty acids rather than carbohydrates [11]. Recent reports have also suggested that increases in body weight and the obesity that results may be the outcome of mitochondrial dysfunction and altered oxidative capacities [11]–[15]. Decreased mitochondrial mass, activity and DNA (mtDNA) copy number have been reported in murine ob/ob, db/db, and diet induced obesity models [16], [17]. Negative correlation between degree/severity of obesity and expression and activity of mitochondrial oxidative phosphorylation (OXPHOS) components have also been observed in human subjects [18]. Lastly, altering mitochondrial activity has been shown to protect mice against diet-induced obesity [19]. Improved understanding of the multiple factors that may contribute to alterations in body weight and obesity development, including the gut microbiota and mitochondrial activity may offer novel insights into key alternative strategies for the treatment of obesity and its associated co-morbidities.

Interleukin-15 (IL-15) is an immunomodulatory cytokine primarily known for its effects on lymphocytes, specifically Natural Killer (NK) cells and CD8+ T cells [20]–[22], and more recently, for its association with obesity and body mass regulation. It has been reported that obese rodents and individuals have lower circulating levels of this cytokine [23]–[25] and treatment with IL-15 induces weight loss in wildtype C57BL/6 (B6) mice [26], IL-15−/− mice, and murine models of obesity [25], [27]. IL-15 has been shown to decrease adiposity, lipid incorporation into adipose tissue, and adipocyte size [25], [26], [28]–[31], as well as affect adipokine adiponectin secretion [32]. Since immune cells may directly induce lipolysis in white adipose tissue [33] and IL-15 affects the survival and activity of lymphocytes [20]–[22], we previously examined the nature of these interactions and found that IL-15 mediated weight loss occurs independent of lymphocytes [26]. Uncovering the potential lipolytic effects of IL-15 on adipose tissue therefore requires examining its potential effects on mitochondrial fatty acid usage. Interestingly, recent reports suggest that IL-15 treatment affects mitochondrial function by increasing the expression of mitochondrial enzymes such as carnitine palmitoyltransferase-1α (*CPT-1α*), which is involved in fatty acid oxidation, in both brown adipose tissue and in CD8+ memory T cells, as well as promotes mitochondrial biogenesis in this T cell subset [30], [34]. As well, IL-15 treatment has been shown to induce uncoupling protein-1 expression in brown adipose tissue [30], skeletal muscle expression of peroxisome proliferator-activated receptor (PPAR)-δ, and significantly increase whole body and skeletal muscle fatty acid oxidation, while decreasing muscle fat content [31]. Altogether, these results suggest that IL-15 treatment may have direct effects on mitochondrial factors involved in beta oxidation in adipocytes. Since IL-15 has been reported as an anabolic factor for skeletal muscle [35], understanding how IL-15 may contribute to the regulation of body weight, adipose tissue, and adipocyte size may reveal novel molecular pathway(s) involved in altering body composition.

In this study, we sought to determine the underlying mechanisms involved in mediating differences in body weight between genetically modified mice having either high levels of IL15 (IL15tg) or no IL15 (IL15−/−). We first determined if varying levels of IL-15 expression altered activity level, food consumption, and respiratory exchange ratio in IL-15tg, B6, and IL-15−/− mice by performing metabolic cage studies. In order to examine the role of the intestinal microbiota in mediating differences in weight, we assessed intestinal bacterial diversity, performed colonization experiments using germ free animals, and evaluated intestinal macronutrient absorption with a glucose and lipid gavage. To determine whether IL-15 has direct effects on adipocytes, we treated mature murine 3T3-L1 adipocytes and assessed its lipolytic effects, genetic induction of mitochondrial fatty acid oxidation expression markers, and adipokine secretion. Lastly, we examined mitochondrial factors in cultured adipocytes and in metabolic tissues of our three strains of mice. Altogether, these experiments will contribute to our understanding of the underlying mechanisms involved in IL-15's effects on body weight and its regulation of adipose tissue.

## Results

### Varying expression of IL-15 does not alter food consumption, water intake, activity level, respiratory exchange ratio, and core body temperature

**Figure 1:**
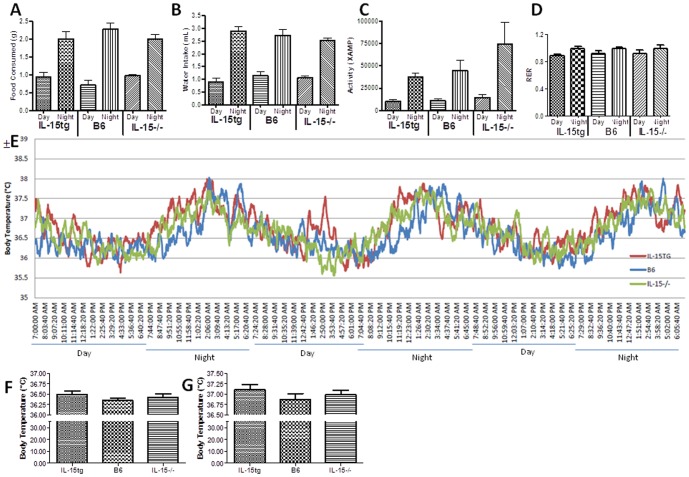
Varying expression of IL-15 does not alter food consumption, water intake, activity level, respiratory exchange ratio, and core body temperature. Bar graphs show (A) food consumption, (B) water intake, and (C) activity levels of 2 month old female IL-15tg (n = 4), B6 (n = 5), and IL-15−/− (n = 5) mice individually housed in metabolic cages (Columbus Instruments Laboratory Animal Monitoring System) for 72 hours. (D) Respiratory exchange ratio (RER) determined by CO_2_ production and O_2_ intake between 3 strains of mice. (E) Line graph shows average core body temperature during the day and night cycle of mice with visceral cavity temperature probes over a 72 hour period. Average (F) day and (G) night body temperatures depicted in bar graphs. Data expressed as mean ± SEM.

To investigate the underlying mechanisms by which IL-15 regulates body mass, we performed metabolic cage studies in two month old female mice prior to differences in body weight among strains. At this time point, there were no significant differences in food intake, water intake, and activity level between IL- 15tg, IL-15−/−, and B6 mice during the day and night cycles (**Fig. 1A-C**). Analysis of respiratory gases showed no differences in respiratory exchange ratios (RER) between our three strains, suggesting similar utilization of macronutrients (**Fig. 1D**). Lastly, there were no significant differences detected in core body temperature among the three strains of mice during the day and night cycles (**Fig. 1E-H**).

### Varying expression of IL-15 alters gut microbiota, but does not alter glucose or lipid absorption

**Figure 2:**
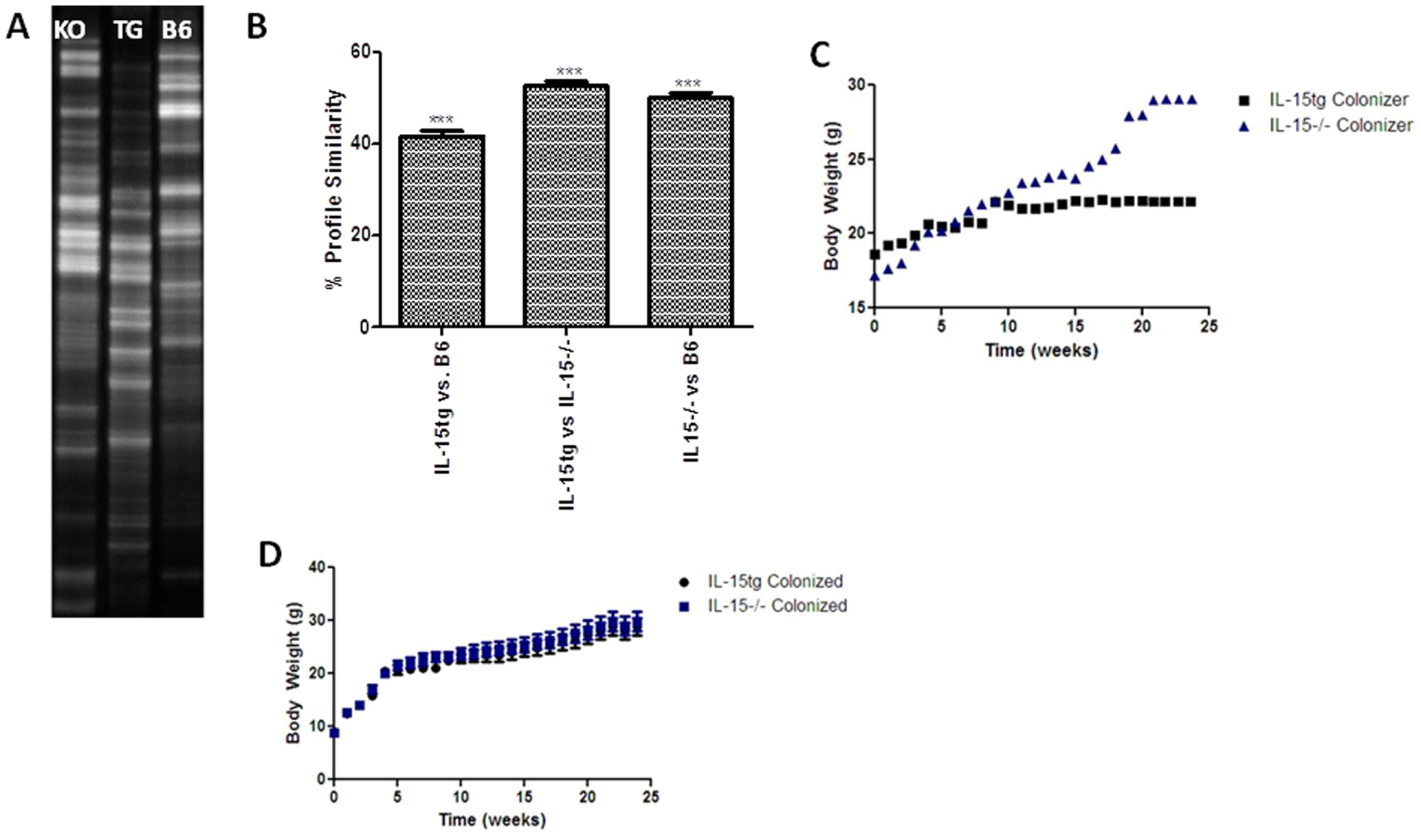
Varying expression of IL-15 alters gut microbiota and does not play a role in modulating body weight. (**A**) PCR-DGGE targeting the v3 (variable region #3) region from bacterial 16S rDNA sequences isolated from IL-15tg (n = 5), B6 (n = 9), and IL-15−/− mice stool samples (n = 4). (**B**) Profile similarity generated from inter-mouse group comparisons (IL15−/− vs IL15tg vs B6). Intra-group comparisons were not significantly different from each other (i.e. mice within each strain). Body weights of (**C**) IL-15tg and IL-15−/− mice colonizers (n = 1/group) and (**D**) colonized germ free C57Bl/6 (n = 4/group) animals over 6 months. Data expressed as mean ± SEM. ***p<0.001.

Since striking variations in gut bacterial diversity exists between obese and lean individuals, [7]–[9], we wanted to determine whether differences in the gut microbiota exist among our three strains of mice. Using 16S DNA sequencing from collected stool samples, we determined that varying IL-15 expression resulted in different gut bacterial diversity among our three strains (**Figs. 2A and 2B**). In order to test whether these alterations mediate differences in body weight, we co-housed germ free animals with either an IL-15tg or IL-15−/− female colonizer and monitored their body weight over time. We found that over time, although the weights of the colonizer's diverged, the body weights of germ free animals colonized with either IL-15tg or IL-15−/− microbiota did not differ significantly suggesting the gut microbiota does not play a role in mediating differences in body weight (**Figs. 2C and 2D**).

**Figure 3:**
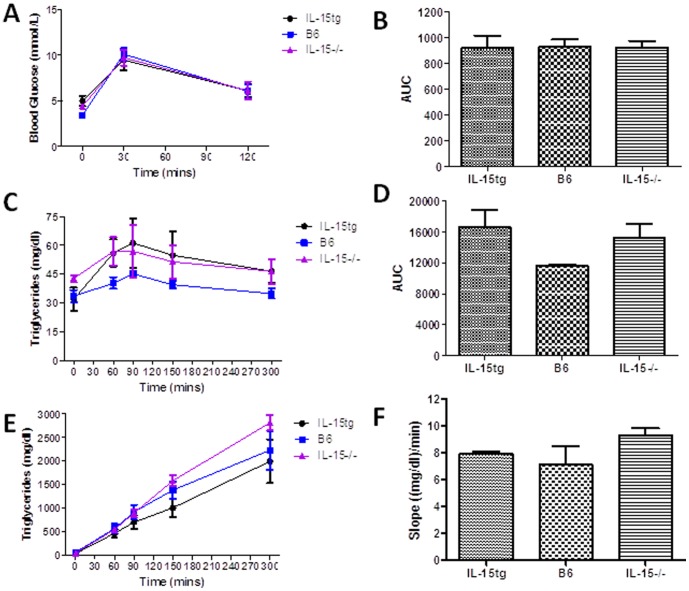
Varying IL-15 expression does not alter glucose or lipid absorption. (**A**) Blood glucose (mmol/l) and triglyceride (mg/dl) concentrations in the (**C**) absence or (**E**) presence of the lipase inhibitor tyoxapol at baseline and at various time points following administration of an oral glucose or olive oil load normalized to body weight in 6 month old female mice. Bar graphs depict area under the curve (AUC) or slope for the total (**B**) glucose and (**D, F**) lipid responses during the oral challenge (n = 5/group). Data expressed as mean ± SEM.

Studies have shown that differences in bacterial diversity in the gut alter macronutrient absorption [36], [37]. As well, since IL-15tg mice are susceptible to autoimmunity, inflammation in the small intestine of these animals may hinder macronutrient absorption and subsequent utilization [38]. To further elucidate potential defects in glucose and fat absorption that could contribute to altered body weight, overnight fasted 2 and 6 month old female IL-15tg, B6, and IL-15−/− mice were orally administered glucose or a lipid load of olive oil normalized to their body weight. Glucose or lipids were administered at these ages to determine if significant differences in glucose or lipid absorption among strains were age dependent. Oral lipids were administered in the presence or absence of the lipase inhibitor tyloxapol. We found no differences in glucose (**Figs. 3A and 3B**) and triglyceride (**Fig. 3C-F**) levels at any time point in both 2 (**S1 Figure**) and 6 month old mice, suggesting that varying levels of IL-15 do not affect intestinal glucose and lipid absorption.

### IL-15 has direct effects on adipocytes and production of IL-6 and KC

**Figure 4:**
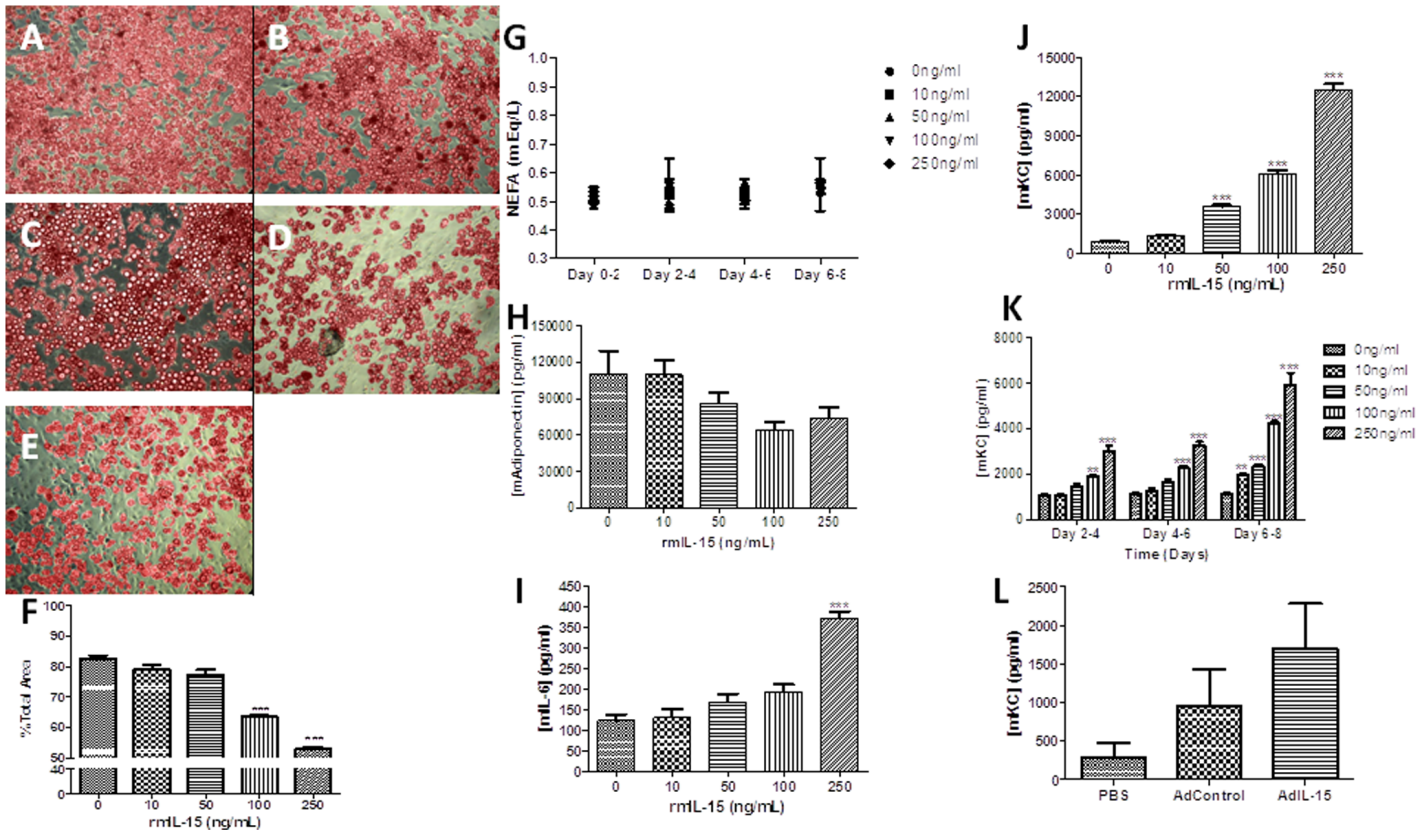
IL-15 treatment results in decreased lipid deposition in 3T3-L1 mature adipocytes and IL-6 and KC production with increasing IL-15 treatment. Mature 3T3-L1 adipocytes were treated every 2 days with either (A) 0, (B) 10, (C) 50, (D) 100, or (E) 250 ng/ml of recombinant murine IL-15 and were stained with Oil Red O. Pictures were taken under a ×10 objective. (F) Relative semi-quantitative differences in lipid deposition were confirmed using ImageJ software (n = 3 images/group). (G) Non-esterified fatty acid concentrations were quantified in 3T3-L1 cell supernatants at each time point (n = 3/group). Differentiated 3T3-L1 adipocytes were seeded at 2×10^5^ cells/well and stimulated in triplicate with the indicated concentrations of rmIL-15 for 24 hours at 37°C. Differentiated 3T3-L1 adipocytes were stimulated in triplicate with the indicated concentrations of rmIL-15 for 24 hours at 37°C and cell supernatants were analyzed for (H) adiponectin, (I) interleukin-6 (IL-6), and (J) keratinocyte-derived chemokine (KC) production by ELISA. Data expressed as mean ± SEM. **p<0.01, ***p<0.001.

Previously, we have shown that AdIL-15 treatment induced cell shrinkage of mature adipocytes in RAG2-/-γ_c_−/− mice at day 8 post-treatment [26]. Whether this is a direct effect of IL-15 has yet to be determined. We and others have shown that IL-15 affects lipid deposition in differentiating human and murine 3T3-L1 adipocytes [25], [32], [39]; however, these experiments examine the effects of IL-15 on adipocytes during differentiation and do not assess its effects on mature adipocytes *in vitro*. Conflicting reports have yet to clarify whether IL-15 can directly mediate lipolysis in mature adipocytes [28], [32]. Therefore we utilized the murine preadipocyte 3T3-L1 cell line to determine if IL-15 directly affects adipocytes. Once terminally differentiated at day 14, murine adipocytes were treated with 0 ng/ml, 10 ng/ml, 50 ng/ml, 100 ng/ml, or 250 ng/ml of recombinant murine (rm) IL-15 protein in triplicate for 8 days with cell supernatants collected every two days. As shown in **Fig. 4A-E**, IL-15 treatment reduced oil red O staining in a dose dependent manner. When the percent of area stained was quantified, adipocytes treated with 100 ng/ml and 250 ng/ml rmIL-15 for 8 days had significantly lower ‘red’ density staining compared to untreated adipocytes (**Fig. 4F**). Non-esterified free fatty acid (NEFA) concentrations were measured in cell supernatants as a marker of lipolysis due to rmIL-15 treatment. **Fig. 4G** shows that IL-15 treatment did not result in increased concentrations of NEFAs in culture supernatants compared to controls at any time point. We also wanted to determine whether short-term rmIL-15 treatment on adipocytes affected adipokine secretion. RANTES, TNF-α, and leptin levels were undetectable. Despite no significant difference in adiponectin production with cytokine treatment (**Fig. 4H**), we found that after 24 hour treatment at various doses, mature adipocytes produce murine IL-6 (**Fig. 4I**) at the highest IL-15 dose and keratinocyte-derived chemokine (mKC) in a dose-dependent fashion (**Fig. 4J**). We then examined IL-6 and KC production over the 8 day time course and found that cytokine treatment induced KC production in a dose and time dependent manner, while IL-6 was undetected from day 2 onwards (**Fig. 4K**). Since IL-15 mediated weight loss in B6 mice [26], we then wanted to correlate this effect with KC and IL-6 *in vivo.* We found no significant difference in circulating KC and no detectable IL-6 among AdIL-15, AdControl, and PBS groups on day 8.

### IL-15 affects adipose mitochondrial membrane potential *in vitro* and mitochondrial mass and activity *in vivo*

**Figure 5:**
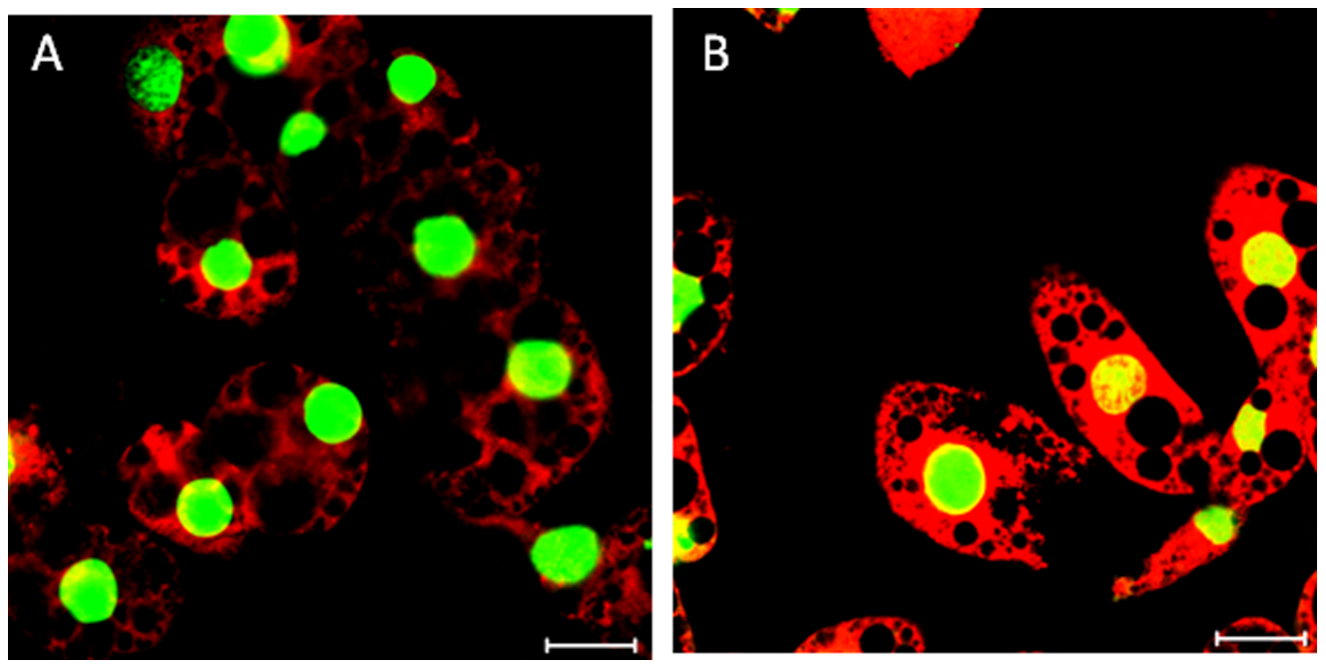
IL-15 treatment increases membrane potential in 3T3-L1 mature adipocytes *in vitro.* Confocal images show fixed mature 3T3-L1 cells either (**A**) untreated or (**B**) treated with 250 ng/ml rmIL-15 for 24 hours. Cells were stained with mitotracker and counterstained with SYTO nucleic acid stain (green). Images were obtained using LSM 510 confocal microscope using ×63 objective. Original magnification is ×1260 and scale bar depicts 20 µm.

**Figure 6:**
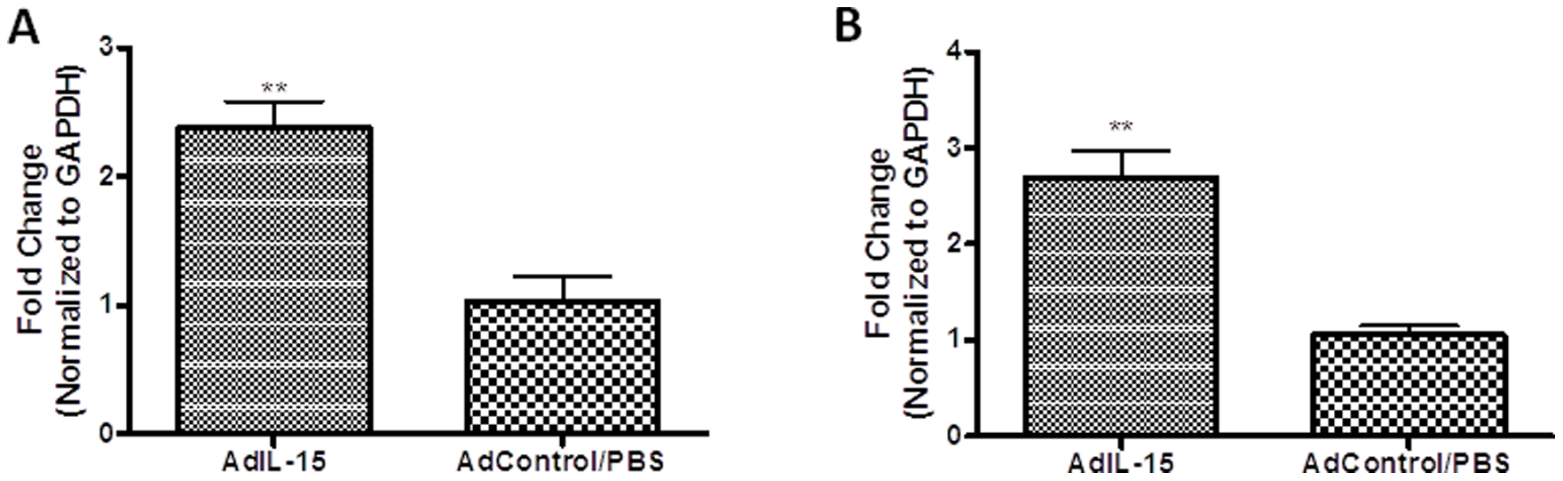
AdIL-15 treatment increases expression of fatty acid oxidation markers *CPT1a* and *CHAD* in adipose tissue. RAG2−/−γc−/− mice were treated with either AdIL-15 or AdControl/PBS on days 0, 2, and 4. RNA was isolated from collected adipose tissue on day 8 and analyzed for genetic expression of (**A**) CPT1a and (**B**) CHAD using RT-PCR. Genetic mRNA expression levels were standardized to internal GAPDH levels and reported as fold increase relative to AdControl/PBS treated animals. (n = 5/group). **P<0.01.

Since differences in body weight have been attributed to altered mitochondrial function, we wanted to determine if IL-15 treatment resulted in altered mitochondrial activity *in vitro* by staining murine adipocytes with Mitotracker. Mitotracker is a mitochondria-specific cationic fluorescent dye used to evaluate mitochondrial membrane potential, which is important for the conversion of ADP-ATP via ATP synthase. When mature 3T3-L1 cells treated with 250 ng/ml rmIL-15 for 24 hours were incubated with Mitotracker, we found that mitochondrial membrane potential was significantly increased compared to control untreated adipocytes (**Fig. 5**). These results suggest that IL-15 has direct effects on mitochondria in adipose tissue. We then wanted to assess IL-15's effects on genes associated with fatty acid oxidation. **S2 Figure** shows genes that were upregulated and downregulated with rmIL-15 treatment *in vitro*. Significant genetic downregulation of genes involved in fatty acid catabolism was seen in acyl-coA dehydrogenase (*Acadsb*), oxidase (*Acox2*), and synthetase (*Ascl3*). *In vivo* analysis shows that AdIL-15 treated RAG2−/−γc−/− mice had an upregulation of mitochondrial fatty acid oxidation markers *CPT1a* and beta-hydroxyacyl CoA dehydrogenase (*CHAD*) in adipose tissue (**Figs. 6A and 6B**). Altogether these results suggest that IL-15 treatment may promote fatty acid oxidation in adipose tissue.

**Figure 7:**
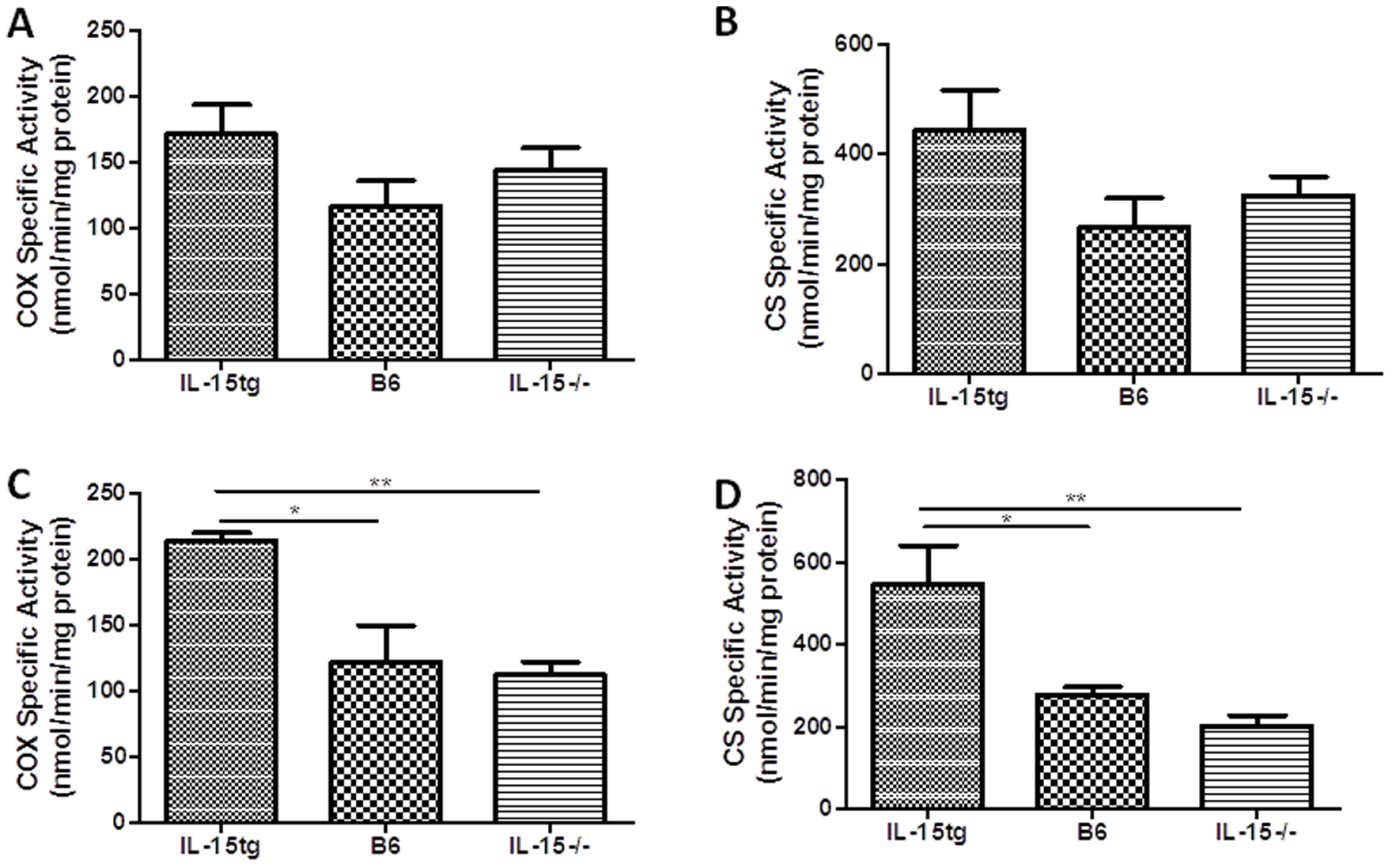
IL-15tg mice have increased mitochondrial activity and mass at 6 months of age in adipose tissue. Adipose tissue homogenates from (**A, B**) 2 and (**C,D**) 6 month old female IL-15tg, B6, and IL-15−/− mice were analyzed for (**A,C**) complex IV activity and (**B,D**) citrate synthase activity. Data are expressed as the mean enzyme activity (nmol/min/mg protein) ± SEM. (n = 5 per group unless otherwise stated; for 6 months of age IL-15tg (n = 4/group) and B6 (n = 4/group)) *P<0.05, **P<0.01.

Lastly, we wanted determine if varying IL-15 expression results in altered mitochondrial function and mass within metabolic tissues of our three strains of mice. Livers, quadriceps muscles, and gonadal fat sources were excised from 2 and 6 month old IL-15tg, B6, and IL-15−/− female mice to determine complex IV (COX) activity, as a measure of mitochondrial activity, and for citrate synthase activity, as a measure of mitochondrial mass [40], [41]. We found that 6 month old, but not 2 month old (**Figs. 7A and 7B**) IL-15tg mice have significantly elevated mitochondrial activity and mass specific to adipose tissue compared to age-matched B6 and IL-15−/− mice (**Figs. 7C and 7D**). Statistical analysis using two way ANOVA revealed no significant interaction between age and murine strain for adipose citrate synthase and COX measurements. These differences were not seen in liver or quadriceps muscle samples at either age (**S3** **and** **S4 Figures**). This data suggest that over time, overexpression of IL-15 results in increased mitochondrial activity and mass in adipose tissue.

## Discussion

Results from this study show that differences in body weight between lean IL-15tg, B6 and overweight IL-15−/− mice are not due to alterations in activity level, food intake, or respiratory exchange ratio. We also discovered that despite vast differences in the gut microbiota profiles among these murine strains, these differences did not affect glucose and lipid absorption, as well as differences in body weight over time in colonized germ free animals. *In vitro* studies using cultured 3T3-L1 adipocytes, demonstrate that IL-15 treatment stimulated the secretion of IL-6 and keratinocyte-derived chemokine (KC) in a dose and time dependent manner, as well as resulted in decreased lipid deposition over time. Lastly, IL-15 treatment in cultured adipocytes resulted in increased mitochondrial membrane potential *in vitro*. *In vivo*, IL-15 administration resulted in increased expression of fatty acid oxidation markers *CPT1a* and *CHAD*, while IL-15tg mice have significantly elevated mitochondrial activity and mass in adipose tissue compared to B6 and IL-15−/− mice.

Previous reports have examined the effect of IL-15 expression on host metabolism. Animal monitoring analyses have been recently conducted in a different, muscle specific murine IL-15tg mouse model [42]. Similar to our previous report [25], the outcome in this model was significant reductions in body fat [29]. Here, researchers found that four month old male IL-15tg mice had significantly lower RER and elevated total and ambulatory activity and energy expenditure compared to B6 control mice. Similar to our results, however, no significant difference in food consumption was found [42]. Unlike the transgenic model examined in this paper, the data from the muscle specific model suggest that overexpression of IL-15 correlated to increased fatty acid utilization and affects whole body energy expenditure and movement. The effects of cytokine overexpression on body composition have been previously reported to be dependent on gender. When comparing male and female IL-15tg mice, females had increased lean muscle mass compared to males, while male mice remained lean when challenged with a high fat diet unlike female mice, who had increased adiposity [29]. These data suggest that gender effects may be evident when examining the metabolic consequences of varying IL-15 expression. Aside from differences in the age and gender of mice tested, substantial variation in the genetic regulation of and circulating IL-15 levels among the transgenic models may also contribute to the dissimilarity in results. With the IL-15tg mouse model utilized here, mature murine IL-15 peptide expression is under the control of the MHC class I D^d^ promoter, with serum IL-15 measured at approximately 187 pg/ml [43]. In the mouse model examined by Quinn *et al*., cytokine expression is driven by the human alpha skeletal actin promoter and the transgene's signal sequence was replaced with that of IL-2, with circulating levels measured at approximately 103,000 pg/ml [29], [42]. Therefore, examining the metabolic consequences of IL-15 overexpression will most likely depend on its genetic regulation, with differences in measured outcomes partially due to global or tissue specific expression, as well as variation in circulating cytokine levels and gender of animals tested.

Along with human studies connecting altered gut microbiota to obesity, it has been previously reported that deficiencies in innate immune factors, such as toll-like receptor-5 (TLR-5), have been associated with significant alterations in gut microbiota and adiposity [44]. Therefore, we wanted to determine if varying IL-15 expression correlated to differences in gut microbiota, and whether this mediated differences in body weight. Although we did not sequence the bacterial species characteristic of each mouse strain, we are the first group to show that varying IL-15 expression results in striking differences in the gut bacterial profile of mice. Significant alterations in the gut microbiota have been shown to affect body weight and other metabolic processes such as bile-acid metabolism and subsequent intestinal lipid absorption, insulin sensitivity, and hepatic steatosis [45]–[49]. In our studies we found that body weight and intestinal lipid and glucose absorption did not differ in our three strains of mice, suggesting that the gut microbiota does not play a role in mediating differences in body weight or affect absorption. A previous study from our lab showed that acute IL-15 treatment in diet induced obese mice improved glucose homeostasis and insulin sensitivity [50]. Although diet induced obesity is characterized by specific gut bacterial changes, whether acute IL-15 treatment in obese animals result in its own characteristic bacterial profiles and mediates the improved glucose and insulin responses have yet to be determined. Since Quinn *et al.* [29] showed that male IL-15tg animals are protected against diet induced obesity, it is possible that the importance of the gut bacterial differences in our three strains of mice become evident when challenged with a high fat, where similar results in improved glucose homeostasis and insulin sensitivity become evident in our IL-15tg compared to B6 and IL-15−/− animals.

The precise role and biological significance of the unique gut bacterial profiles in our three strains of mice is unknown. These bacterial differences in mice with various IL-15 expression levels may exist as a consequence of the disparities in the intestinal immune and inflammatory status, due to the influence of IL-15. Another possibility is that since the gut microbiota is a rich source of molecules that may cause inflammation, like lipopolyssaccharide and peptidoglycan [51], differences in the bacteria present may play a role in contributing to the intestinal inflammatory environment and/or gut permeability via effects on the gut epithelium. Research has shown that IL-15 expression plays a role in mediating intestinal inflammation in models of celiac disease and Crohn's disease [38], [52]–[54], suggesting a possible link between IL-15, gut microbiota, and intestinal inflammation. Defining the metabolic contribution of the gut microbiota in our three strains of mice is currently under investigation.

The results of this paper demonstrate that IL-15 has direct effects on adipocytes *in* vitro through the secretion of adipokines and reduction in adipocyte lipid content. The secretion of proinflammatory adipokines IL-6 and KC reported here, and of the anti-inflammatory adipokine adiponectin reported by Quinn *et al*. [32] by adipocytes after 4–6 days of treatment, may affect the inflammatory status of adipose tissue by IL-15 administration *in vivo*. Similar to Quinn's results, we did not find any significant adiponectin production within the first 24 hours of IL-15 treatment [32]. Due to IL-6's known lipolytic effects [55], we wanted to determine how IL-6 production correlated with IL-15 treatment *in vivo* and *in vitro*. These results suggest that IL-6 may play a role in IL-15 mediated lipolysis. The use of neutralizing antibodies against IL-6 would determine whether IL-15 requires IL-6 expression to induce weight loss *in vivo* and its effects on cultured adipocytes. Since IL-6 was detected *in vitro* only 24 hours after IL-15 treatment, we believe we were unable to detect serum IL-6 on day 8 in AdIL-15, AdControl, and PBS treated animals due to differences in when samples were collected. If blood samples were collected 24 hours post treatment, we may have detected similar differences in serum IL-6 levels of AdIL-15 treated animals that would then match our *in vitro* data.

In regards to the induction of KC production, at this time the authors are not aware of any published findings demonstrating this chemokine's possible lipolytic properties. We found that KC was detected *in vitro* and *in vivo* at day 8; however, we found significant differences in cell culture treatments while our *in vivo* data reflected the *in vitro* data trend but was not significant. By increasing our numbers per treatment group, as well as taking multiple blood time points during the 8 day period, we could determine whether KC production peaks at a different time point *in vivo* and if the groups differed significantly. Murine KC is the homolog to human IL-8. Increased KC expression is found in adipose and plasma of ob/ob and diet induced obese mouse models, and is primarily produced by stromal vascular cells. Interestingly, KC is highly expressed by preadipocytes and decreases during adipogenesis. Although KC has no effect on adipogenesis, whether this chemokine has effects on mature adipocytes has yet to be determined [56].

In this study, we are also the first to show that IL-15tg mice have increased mitochondrial activity and mass specific to adipose tissue at 6 months of age compared to B6 and IL-15−/− mice. Whether these mitochondrial alterations precede and mediate changes in body weight, or result from body weight differences have yet to be determined. At the 2 month time point (**Figs. 7A and 7B**), mitochondrial activity and mass appear to be slightly elevated in the adipose tissue of IL-15tg mice, suggesting mitochondrial alterations may precede differences in body weight. Previous reports suggest that deficiencies in IL-15 signaling (IL-15Rα−/− mice) increased skeletal muscle mitochondrial biogenesis, density, expression of complexes involved in the electron transport chain, and mitochondrial DNA content [57], [58]. As well, muscle specific male IL-15tg mice had increased mRNA and protein expression of factors involved in promoting lipid oxidation in extensor digitorum longus muscles compared to control mice. The increased expression of skeletal muscle oxidation markers in muscle specific IL-15tg mice correlated to a significant increase in the duration of a single run-to-exhaustion endurance test compared to B6 controls [42]. However, we found no significant differences in quadriceps muscle mitochondrial mass and activity at either time point between IL-15−/−, B6, and IL-15tg mice.

We also show that IL-15 treatment increased mitochondrial membrane potential in cultured adipocytes when stained with Mitotracker. Since IL-15 treatment caused significant reduction in lipid content over time, we wanted to determine if this increase in mitochondrial membrane potential correlated to the induction of fatty acid oxidation by examining various genetic markers involved in this process. Whether this increase in mitochondrial membrane potential is specific to fatty acid oxidation is not certain in our *in vitro* model since no significant upregulation in these markers was found after 24 hours of treatment (**S2 Figure**). However, our *in vivo* analysis demonstrates that IL-15 increases genetic expression of fatty acid oxidation markers such as *CPT1a*, an enzyme involved in mitochondrial fatty acid transport, and *CHAD*, an enzyme involved in beta oxidation. Similarly, other reports have shown that IL-15 administration resulted in increased *CPT1a* expression in brown adipose tissue and in CD8+ memory T cells [30], [34].

In order to produce adenosine-5′-triphosphate (ATP), breakdown of pyruvate and fatty acids must occur in the mitochondria through glycolysis and the Krebs cycle. Reduced intermediates are then shuttled through the electron transport chain, which generates the majority of the cell's ATP. The transport of electrons results in a potential gradient across the mitochondrial inner membrane, which is essential for protons to enter the mitochondrial matrix resulting in ATP production [59]. Since IL-15 treatment affects mitochondrial membrane potential, this suggests that cytokine treatment could directly affect the potential gradient across the mitochondrial inner membrane and therefore the electron transport chain, subsequently affecting ATP synthase activity and ATP production. Determining intracellular ATP production in adipocytes treated with IL-15 will determine whether IL-15 affects ATP production. Altogether, this suggests that IL-15 mediates its effects directly on adipocytes through the induction of lipolysis and subsequent oxidation of fatty acids, leading to increased ATP production.

In summary, our findings suggest that IL-15 directly affects adipocytes through adipokine secretion of KC and IL-6, reduction in lipid content, and increases mitochondrial membrane potential *in vitro*. Our animal studies suggest that IL-15 expression alters mitochondrial activity and mass and induces genetic expression of fatty acid oxidation markers *in vivo*, which could modulate body composition. Further work is needed in order to define the biochemical and signaling mechanism(s) of IL-15 action on adipose tissue, as well as its effects on other metabolic tissues to mediate these alterations in body composition. Similar to the approach of this paper, examining a variety of factors that may alter body weight is necessary in order to thoroughly understand the importance and relative contribution of each, which will lead to improved therapeutic and preventive strategies for obesity.

## Materials and Methods

### Ethics

All animal experiments were approved by the Animal Research Ethic Board (AREB) of McMaster University. The AREB approval number is: 10-02-12.

### Mice

A breeding pair of IL-15tg mice on a C57BL/6 background was kindly provided by M. Caligiuri (Ohio State University, School of Medicine, Columbus, OH), while breeding pairs of C57BL/6 and IL-15−/− mice were purchased from Charles River Laboratory (Montreal, Quebec, Canada) and Taconic (Germantown, NY, USA), respectively. Female lymphocyte deficient Balb/c RAG-2−/−γc−/− mice were bred from breeding pairs given as a gift by M. Ito (Central Institute for Experimental Animals, Kawasaki, Japan). These three strains of mice were bred and maintained at McMaster University's Central Animal Facility. All experiments were approved by the Animal Research Ethics Committee. Mice were housed in level B rooms and maintained under controlled lighting (12 hour light/12 hour dark cycle) and temperature (22°C) with ad libitum access to water and a low 5% fat chow diet. At 2 months of age, metabolic monitoring was performed using a Comprehensive Lab Animal Monitoring System (Columbus Instruments, Columbus, OH) as previously described [60]. Core body temperature was assessed by visceral implantation of TA-F10 mouse temperature transmitters and analyzed using ART v4.3 software (Data Sciences International, St. Paul, Minnesota) over a 72 hour period.

### Bacterial DNA determination of gut microbiota

Bacterial DNA was extracted from stool samples collected from 2 month old female IL-15tg, B6, and IL-15−/− mice using the QIAamp DNA Stool Mini Kit (QIAGEN, Toronto, Ontario, Canada) and quantified spectrophotometrically. Amplification of the hypervariable V3 region from the bacterial 16S ribosomal DNA using polymerase chain reaction was completed with universal bacterial primers (HDA1-GC,HDA-2; Mobixlab, McMaster University core facility, Hamilton, Ontario, Canada) as previously described [61]. Denaturing gradient gel electrophoresis (DGGE) was performed using a DCode universal mutation system (Bio-Rad, Mississauga, Ontario, Canada) and the electrophoresis was conducted at 130 V, 60°C for 4.5 hours. Gels were stained with SYBR green I (Sigma, St. Louis, MO, USA) and viewed by ultraviolet transillumination. A scanned image of the electrophoretic gel was used to measure the staining intensity of the fragments using Quantity One software generating an electrophoregram (version 4-2; Bio-Rad Laboratories). The staining intensity of each fragment was compared using the Dice similarity coefficient [61].

Two month old female germ-free mice acquired from McMaster University's gnotobiotic unit, were co-housed with either a two month old female IL-15tg or IL-15−/− mouse and their body weights were monitored on a weekly basis during a 6 month period.

### Glucose and triglyceride gavage

At both 2 and 6 months of age, female IL-15tg, B6, and IL-15−/− mice were administered 2 g/kg D-glucose or 10 µl/g olive oil following an overnight fast. The olive oil gavage was performed in either the absence or presence of the lipase inhibitor tyoxapol (Sigma, St. Louis, MO, USA), where mice were administered 0.5 mg/g intravenously immediately after gavage. Blood samples were collected by submandibular bleeds at baseline (time 0) and at various time points post-gavage from each mouse per group. Blood glucose was measured using a hand-held glucometer (Accu-Chek Active, Roche Diagnostics, Laval, Canada). Following the olive oil gavage, blood was passed through heparinized micro-hematocrit capillary tubes (Fisher Scientific, Pittsburgh, PA, USA), collected in eppendorf tubes, and centrifuged at 12,000 rpm for 10 minutes at 4°C to collect plasma. Triglyceride plasma levels were measured using the L-type triglyceride M colorimetric assay (Wako Diagnostics, Richmond, VA, USA). The area under the curve for the total glucose and triglyceride responses was assessed using the trapezoidal rule. Linear regression analysis was used to calculate slopes for triglyceride responses in tyoxapol treated mice.

### Cell culture

Murine preadipocyte 3T3-L1 cells were purchased from ATCC and grown in Dulbecco's Modified Eagle Media (DMEM) supplemented with 10% bovine serum (Invitrogen, Burlington, Ontario, Canada). Cells were seeded at 200,000 cells/well in a 24 well plate and grown/cultured until confluent. Twenty four hours later, confluent cells were treated with adipogenic induction media, defined as day 0, which includes 10% fetal bovine serum (FBS), 10 µg/mL human insulin (Invitrogen, Burlington, Ontario, Canada), 250 nM dexamethasone, and 0.5 mM 3-isobutyl-1-methylxanthine (IBMX) in DMEM for 48 hours (Sigma, St. Louis, MO, USA). The cells were then given 10% FBS DMEM post-induction media for another 12 days. During this time, cells were given fresh media every 2–3 days. Once terminally differentiated at day 14, adipocytes were treated with 0 ng/ml, 10 ng/ml, 50 ng/ml, 100 ng/ml, or 250 ng/ml of recombinant murine (rm) IL-15 protein (Peprotech, Rocky Hill, NJ, USA) in triplicate for 8 days with cell supernatants collected every two days. Non-esterified free fatty acid (NEFA) (Wako Diagnostics, Richmond, VA, USA) concentrations were quantified from collected cell supernatants. After 8 days of treatment (day 22), lipid droplets from treated and untreated adipocytes were stained with Oil Red O as previously described [32] and photographed. Images were then loaded into ImageJ v1.45 s where the ‘red’ density area was approximated by varying the threshold tool. This area and the entire image section were measured and represented as the percentage of red staining from the total area.

### Delivery of IL-15

Sixteen week old female B6 and RAG-2−/−γc−/− mice were administered either 5×10^8^ pfu of the Ad-expressing human IL-15 vector Opt.hIL-15 (“AdIL-15”), an empty adenoviral vector (“AdControl”), or 200 µl PBS via IV tail injections on days 0, 2, and 4 (n = 5 per group) as previously described [62]. On day 8, blood was collected from anaesthetized B6 mice through the abdominal aorta and centrifuged at 5,000 rpm for 10 minutes to collect serum. On day 8, adipose tissue was collected from RAG-2−/−γc−/− mice, homogenized in TRIzol, and stored at −80°Celsius (Invitrogen, Burlington, Ontario, Canada) as per manufacturer's instructions.

### ELISAs for murine leptin, adiponectin, RANTES, TNFA, IL-6 and KC

Cell supernatants were collected from differentiated 3T3-L1 adipocytes treated with 0 ng/ml, 10 ng/ml, 50 ng/ml, 100 ng/ml, and 250 ng/ml of rmIL-15 for 24 hours and examined for murine leptin, adiponectin, regulated on activation normal T cell expressed and secreted (RANTES), tumor necrosis factor-α (TNF-α), interleukin-6 (IL-6), and keratinocyte-derived chemokine (KC) using DuoSet ELISA kits (R&D Systems, Minneapolis, MN, USA).

### Confocal microscopy

Murine 3T3-L1 cells were grown and differentiated into adipocytes on glass cover slips in a 24 well plate. At day 14, adipocytes were then treated with 0 ng/ml or 250 ng/ml rmIL-15 for 24 hours and incubated with 500 nM of Mitotracker Red CMXRos (Invitrogen, Burlington, Ontario, Canada) for 30 minutes. Cells were then fixed with 4% PFA and counterstained for nuclei with propidium iodide and attached to microscope glass slides using Vectasheild hard-set mounting medium (Vector, Burlington, Ontario, Canada). Images were captured by LSM510 (inverted) confocal miscroscope (Zeiss, Oberkochen, Germany) under an ×63 objective and analyzed using LSM 510 version 3.2 software (Zeiss, Oberkochen, Germany).

### Mitochondrial enzyme activity

Livers, quadriceps muscles, and gonadal fat were excised from 2 and 6 month old IL-15tg, B6, and IL-15−/− female mice, flash frozen in liquid nitrogen, and stored at −80°C until homogenized. Tissue samples were homogenized in homogenization buffer (100 mM KCl, 220 mM mannitol, 70 mM sucrose, 1 mM EGTA, and 5 mM HEPES pH 7.4), centrifuged at 600xg for 5 minutes, and supernatants were collected and used to determine complex IV activity, as a measure of mitochondrial activity, and for citrate synthase activity, as a measure of mitochondrial mass [40], [41]. Both assays were performed as previously described using UV-spectrophotometry and the data presented are expressed as the mean enzyme activity (nmol/min/mg protein) relative to the wet weight of tissue [40], [63], [64].

### Assays of mRNA expression

RNA was isolated from adipose tissue of AdIL-15, AdControl, or PBS treated RAG2−/−γc−/− mice on day 8, and from differentiated murine 3T3-L1 adipocytes treated with 0 ng/ml or 500 ng/ml rmIL-15 for 24 hours, purified on RNeasy spin columns (QIAGEN, Toronto, Ontario, Canada), and treated with deoxyribonuclease and genomic DNA removal buffer. Then, 400 ng of RNA was reverse transcribed into cDNA (RT^2^ First Stand Kit; QIAGEN, Toronto, Ontario, Canada). Real-time (RT)-PCR was performed using the RT^2^ Fatty Acid Metabolism PCR array purchased from QIAGEN as per manufacturer's instructions using treated and nontreated 3T3-L1 adipocytes. Primer sets used for RT-PCR murine adipose tissue analysis include: *CPT1a* forward and reverse 5′-GCTGGGCTACTCAGAGGATG-3′ and 5′-CACTGTAGCCTGGTGGGTTT-3′; and *CHAD* forward and reverse 5′-ACCAAACGGAAGACATCCTG-3′ and 5′-AGCTCAGGGTCTTCTCCACA-3′. Preliminary experiments conducted by our lab have shown no statistical differences in the expression of these markers between AdControl and PBS treated tissues and are therefore combined as one group (“AdControl/PBS”). Cycle threshold (C_T_) values were set identically for all plates, sample C_T_ values were normalized to GAPDH C_T_, and fold change of gene expression between untreated controls and treated groups were calculated.

### Statistics

Greater than two group comparisons were made using one-way ANOVA followed by Tukey's post hoc multiple comparisons test with Graph Pad Prism 4 software (La Jolla, CA, USA). Two-way ANOVA followed by Bonferroni post tests were used to determine possible strain and time effects on mitochondrial COX and CS measurements. Student t-test was used for two group comparisons. Results are expressed as mean ± SEM. To compare the intestinal microbiota, the similarity between DGGE profiles was determined using the Dice similarity coefficient and tested for significance using Quantity One software (Biorad, Mississauga, Ontario). Significance is indicated when p<0.05.

## Supporting Information

S1 Figure Varying IL-15 expression does not alter glucose or lipid absorption at 2 months of age. **(A)** Blood glucose (mmol/l) and **(C)** triglyceride (mg/dl) concentrations at baseline and at various time points following administration of an oral glucose or olive oil load, normalized to body weight in 2 month old female mice. Bar graphs depict area under the curve (AUC) for the total **(B)** glucose and **(D)** lipid responses during the oral challenge (n = 5/group). Data shown are representative from one of two separate experiments with similar results.
(TIF)

S2 Figure Acute IL-15 administration results in differential expression of fatty acid oxidation markers. Comparative expression of fatty acid oxidation genes from mature 3T3-L1 adipocytes treated with 0 ng/ml or 500 ng/ml rmIL-15 for 24 hours normalized to GAPDH reveals a 1.2 fold difference or greater using PCR arrays (n = 3/group). *P<0.05.
(TIF)

S3 Figure Mitochondrial activity and mass at 2 months of age in liver and quadriceps muscle. **(A,C)** Liver and **(B, D)** quadriceps muscle homogenates from 2 month old female IL-15tg, B6, and IL-15−/− mice were analyzed for **(A,B)** complex IV activity and **(C,D)** citrate synthase activity. Data are expressed as the mean enzyme activity (nmol/min/mg protein) (n = 5/group).
(TIF)

S4 Figure Mitochondrial activity and mass at 6 months of age in liver and quadriceps muscle. **(A,C)** Liver and **(B, D)** quadriceps muscle homogenates from 6 month old female IL-15tg, B6, and IL-15−/− mice were analyzed for **(A,B)** complex IV activity and **(C,D)** citrate synthase activity. Data are expressed as the mean enzyme activity (nmol/min/mg protein). (IL-15tg and B6 (n = 4/group), IL-15−/− (n = 5/group)) *P<0.05.
(TIF)
