# Supplementary figures and images for: Interleukin-15 Modulates Adipose Tissue by Altering Mitochondrial Mass and Activity
Source: PLoS One. 2014 Dec 17;9(12):e114799. doi: 10.1371/journal.pone.0114799 (PMC4269394; doi:10.1371/journal.pone.0114799)

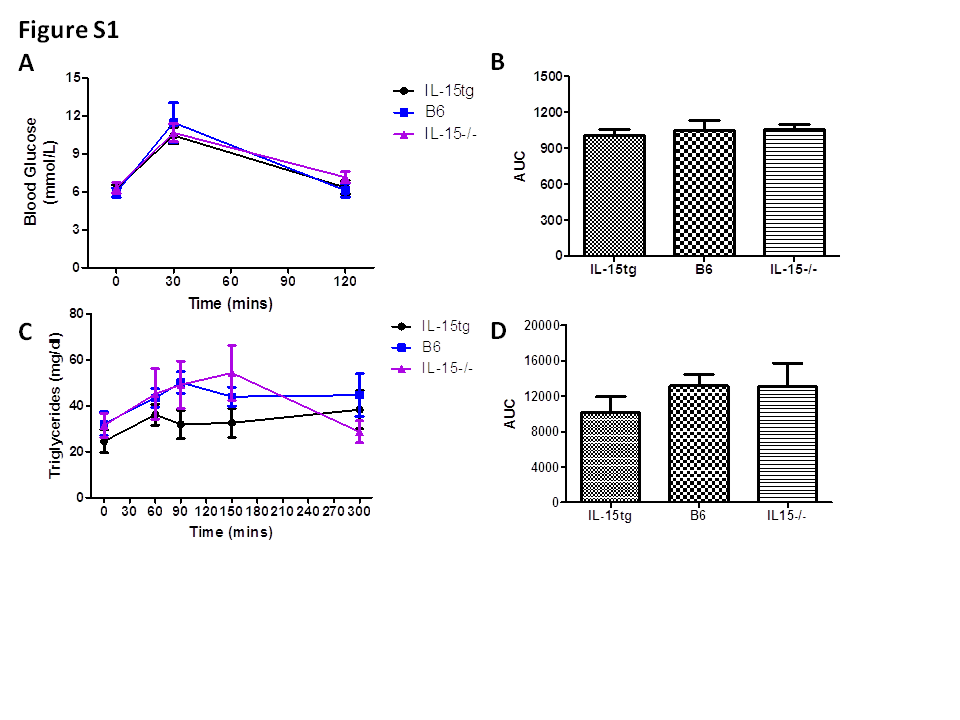

Supplement: S1 Figure — Varying IL-15 expression does not alter glucose or lipid absorption at 2 months of age. (A) Blood glucose (mmol/l) and (C) triglyceride (mg/dl) concentrations at baseline and at various time points following administration of an oral glucose or olive oil load, normalized to body weight in 2 month old female mice. Bar graphs depict area under the curve (AUC) for the total (B) glucose and (D) lipid responses during the oral challenge (n = 5/group). Data shown are representative from one of two separate experiments with similar results. (TIF) [file pone.0114799.s001.tif]

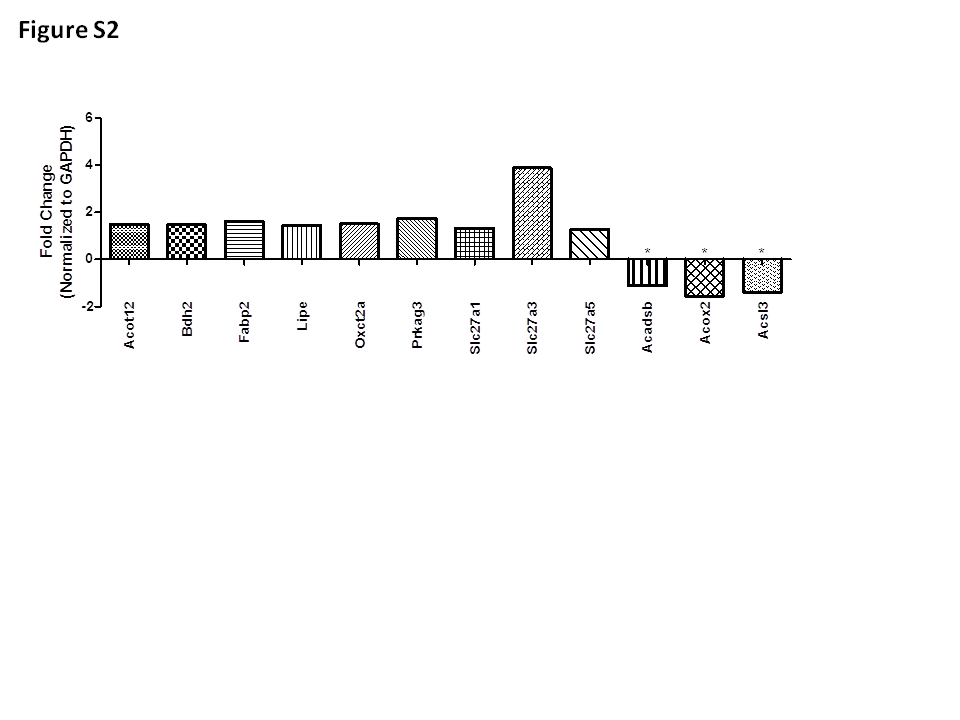

Supplement: S2 Figure — Acute IL-15 administration results in differential expression of fatty acid oxidation markers. Comparative expression of fatty acid oxidation genes from mature 3T3-L1 adipocytes treated with 0 ng/ml or 500 ng/ml rmIL-15 for 24 hours normalized to GAPDH reveals a 1.2 fold difference or greater using PCR arrays (n = 3/group). *P<0.05. (TIF) [file pone.0114799.s002.tif]

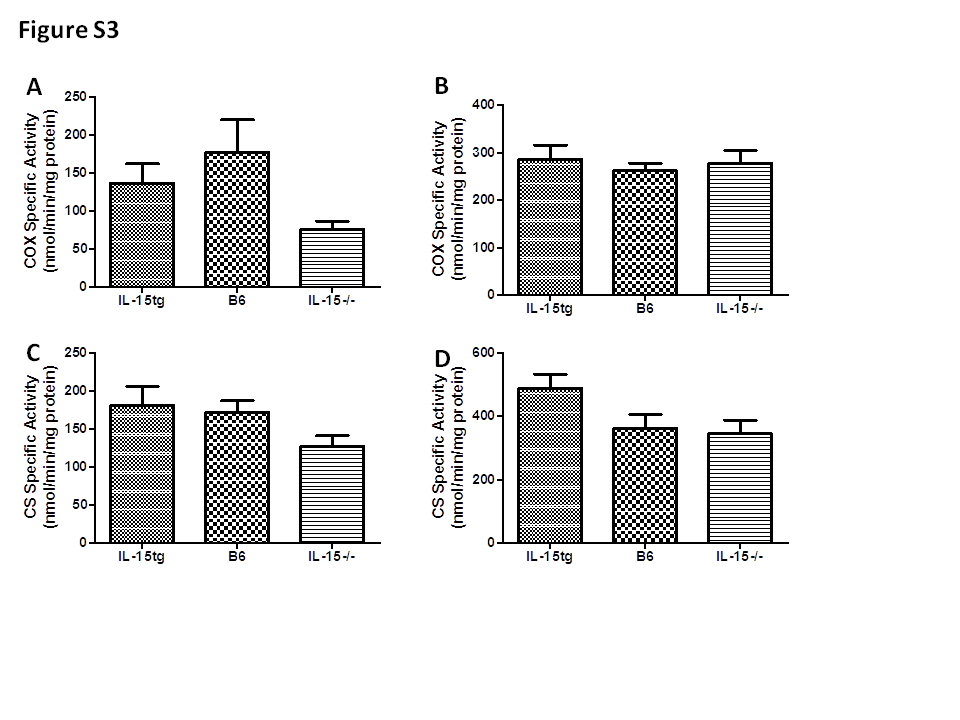

Supplement: S3 Figure — Mitochondrial activity and mass at 2 months of age in liver and quadriceps muscle. (A,C) Liver and (B, D) quadriceps muscle homogenates from 2 month old female IL-15tg, B6, and IL-15−/− mice were analyzed for (A,B) complex IV activity and (C,D) citrate synthase activity. Data are expressed as the mean enzyme activity (nmol/min/mg protein) (n = 5/group). (TIF) [file pone.0114799.s003.tif]

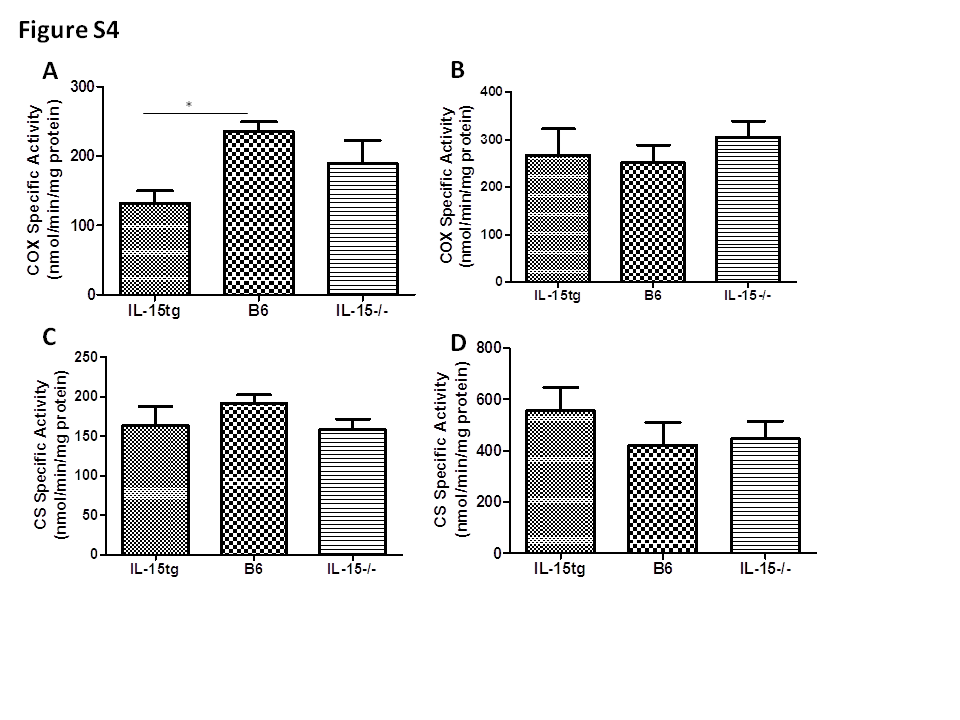

Supplement: S4 Figure — Mitochondrial activity and mass at 6 months of age in liver and quadriceps muscle. (A,C) Liver and (B, D) quadriceps muscle homogenates from 6 month old female IL-15tg, B6, and IL-15−/− mice were analyzed for (A,B) complex IV activity and (C,D) citrate synthase activity. Data are expressed as the mean enzyme activity (nmol/min/mg protein). (IL-15tg and B6 (n = 4/group), IL-15−/− (n = 5/group)) *P<0.05. (TIF) [file pone.0114799.s004.tif]
